# Atomistic-geometric simulations to investigate the mechanical stability of monocrystalline sI methane hydrates under pressure

**DOI:** 10.1038/s41598-023-29194-8

**Published:** 2023-02-02

**Authors:** Xiaodan Zhu, André Guerra, Phillip Servio, Alejandro D. Rey

**Affiliations:** Department of Chemical Engineering, McGill University, Montreal, QC H3A 0C5 Canada

**Keywords:** Mechanical properties, Chemical engineering, Density functional theory

## Abstract

Gas hydrate mechanical stability under pressure is critically important in energy supply, global warming, and carbon-neutral technologies. The stability of these polyhedral guest–host crystals under increasing pressure is affected by host cage type and face connectivity as well as guest gas occupancy. The geometry-imposed cage connectivity generates crystal lattices that include inclusion-matrix material composite structures. In this paper, we integrate Density Functional Theory simulations with a polyhedral-inspired composite material model that quantifies stability limits, failure modes, and the impact of the type of cage occupancy. DFT reveals the existence of two failure mechanisms under increasing pressure: (i) a multistep lattice breakdown under total occupancy and under only large cage occupancy and (ii) a single-step breakdown under zero occupancy as well as with only small cage occupancy. The DFT-composite model predicts optimal occupancy pathways to generate strength and critical occupancy pathways to promote decomposition.

## Introduction

Energy production and sustainability have been critical in the past century. An increasing population, diminishing natural resources, and climate change continue to generate challenges and opportunities for high-energy storage materials. Gas hydrates (GH) are hydrogen-bonded polyhedral guest–host materials that have the versatility to store gases critical to energy supply and climate change and yet be formed by water^1^. Energy supply GH includes sI methane and sII hydrogen, where the distinction (sI, sII) refers to the polyhedral cage types and volume fraction in a crystal lattice.

The stability of gas hydrates is crucial and serves as the foundation for all exploration and research. Natural gas hydrates occur in subsea and artic reservoirs on Earth and contain a substantial amount of methane, which has more than 25 times the effect of carbon dioxide on climate change^1^. Consequently, the first application of the results from this work is to inform whether the hydrate structure is mechanically stable and that no methane is escaping during exploration. Specifically, in recent years, global warming and geological movements have led to the instability of natural gas hydrates and the occurrence of methane seepage in some natural gas hydrate reservoirs. Consequently, the study of hydrate stability can help predict hydrates' resistance to climate change and to crustal movements.

Applications to climate change and global warming include CO_2_ sI GHs that in principle, could be coupled to methane GHs to produce a dual energy-source-and-carbon-sink material system. Currently, hydrogen GHs are being intensely researched and developed due to the opportunity of creating an energy platform of green storage for green hydrogen. Further progress in these energy sources, impact and storage requires a quantitative understanding of mechanical stability limits and failure mechanisms under increasing stress loads and different guest gas compositions and degree of occupancy. In this work, we focus on the mechanical stability of monocrystal methane sI gas hydrates under pressure loads to establish the impact of gas cage occupancy and cage connectivity on failure thresholds and failure modes by integrating atomistic DFT simulations with geometric modelling inspired by composite materials.

Previous research on the stability of gas hydrates based on Density Functional Theory (DFT) and Molecular Dynamics (MD) simulations shed light on various aspects of single cage or lattice scale phenomena. Vlasic et al.^2^ examined the stability of sII hydrates using the Equation of State (EOS) (Murnaghan, Birch-Murnaghan, Vinet, Liu) and the impacts of various guest molecules on the material properties of the hydrates. They discovered that the hydrogen bond density, which serves as the lattice's sustaining force, and the bulk modulus were shown to have a positive linear connection. However, the bulk modulus drops as the atomic volume rises. Additionally, it was discovered that the larger gas molecules would exert an outward force on the cages due to van der Waals repulsions, growing the cages' dimensions and thereby growing the lattice volume. Daghash et al.^3–5^ have made a detailed study on the sH gas hydrates in terms of stability. They reported physical properties at the atomistic level using DFT and quantified the dispersion forces^3^. Also, Daghash et al.^4^ and Vlasic et al.^6^ used a DFT-based IR spectrum to identify the hydrogen bond’s vibration frequency, which can be used to determine the hydrate’s Young modulus and provide a better understanding on the host molecular vibrations. Since the vibrational frequency depends on the pressures and bond length, they have presented a new route for mechanical properties determination. In addition, Daghash et al.^5^ reported the positive linear relationships between the elastic constants and pressures, which implies that the hydrates’ physical properties would change and can be quantified under pressures. Mathews et al.^7^ examined the thermodynamic parameters of the sI hydrate at various temperatures and came to the apparent consensus that, at lower temperatures, DFT can provide more accurate results than MD. By utilizing the DFT, Jendi et al.^8^ discovered that the tensile and compressive ideal strengths of sI pure methane gas hydrates are − 1.10 GPa and 90 GPa, respectively. Jia et al.^9^ found significant correlations between the second-order elastic constants and pressures, and the piezo effects may differ depending on the type of guest molecules. Mirzaeifard et al.^10^ computed the water-methane surface tension by employing pressure and temperature calculations consistent with standard scaling concepts. Guerra et al.^11^ investigated the effects of temperature and pressure on the viscosities of methane and carbon dioxide hydrates. They discovered that temperature impacts are often one order of magnitude greater than piezo-viscous effects. Furthermore, pressure had a one-order-of-magnitude more significant influence on the viscosity of carbon dioxide hydrate systems than on methane hydrate systems. Also, Guerra et al.^12^ showed that by using the Tip4p/ice water model and the OPLS-AA methane model, molecular dynamics (MD) simulations overestimated the experimental data of sI methane hydrate viscosity under pre-nucleation conditions by 84% on average across all conditions examined. Guerra et al.^13^ similarly demonstrated through MD simulations an overestimation of the viscosity of carbon dioxide hydrate systems of 65% on average across all conditions examined for the three CO_2_ force field considered: EPM2, TraPPE, and Zhang. Wu et al.^14^ showed that the natural gas hydrates would be further destabilized and beyond the conventional thermodynamic instability if there is some force-induced ground deformation based on the deformation-induced hydrate dissociation they discovered. Zhu et al.^15,16^ work at the stability of the sI methane hydrate under pressures. They stated that the piezo effect might be seen from the perspectives of electron clouds, atoms, cages, and lattices^16^, along with the values of second-order elastic constants with pressure restrictions^15^. Also, they confirmed that the sI methane hydrates obey the law of mixture^16^ in the calculation of the structure’s bulk modulus.

The standard for analyzing the piezo effect on the mechanics of gas hydrates involves an examination of the second-order elastic constants, the bulk modulus, the Young modulus and other properties. All these studies, however, are premised on the assumption that forces or energy are distributed uniformly throughout the lattice and that the system is within the stability limits. Within the stability limits, a coarse-grained law of the binary mixture method was successfully implemented under full occupancy for methane GH. To resolve the effect of magnitude (volume fraction effect) and type (small cage and/or large cage) of gas occupancy, a ternary component model is necessary since a crystal lattice has three interacting components: the hydrogen bond network, the small polyhedral cages and the large polyhedral cages. After recognizing these three components, the next step is to introduce polyhedral connectivity which together with cage number density defines a cage type as an inclusion or as a continuous matrix and leads to laws of mixtures for material stability under variable composition and cage type occupancy. The three key phenomena addressed in this paper are: (1) what are the stability limits of methane GH under increasing pressure under variable composition and cage occupancy? (2) what are the possible failure mechanisms under various composition and cage occupancy limiting modes? (3) find the stability surface as a function of cage occupancy and the critical steepest occupancy curves that characterize optimal reinforcement and maximal failure conditions.

This work is on monocrystal methane sI stability and failure modes under pressure, and no phase transitions to other hydrates are considered. DFT simulations are at 0 Kelvin. For each DFT simulation, the cage occupancy is fixed until ultimate failure conditions. To clarify, the only situations in this paper that involve thermodynamic stability are the DFT simulations. This is because, under the given conditions, DFT simulations would relax the system to its lowest-energy state, which is the thermodynamic equilibrium. However, stability limits refer to mechanical stability limitations. It represents the maximum pressure the system can withstand before being destabilized by mechanical forces. Since this work focuses on investigating the piezo effect on structural stability, the research of thermodynamic stability is beyond the scope of this work. The stability surface (Monge patch^17^) is derived from a ternary law of mixtures taking into account the first-order cage-cage interaction nonlinearity. Additionally, computational work involving gas hydrates is benefitted by the integrated use of experimental data^18^. However, to the best of our knowledge, experimental data relevant to the analysis performed here is not available in the literature. Conditions, processes, and mechanisms outside those mentioned above are outside the scope of this paper and can be included in future work as experimental data at the relevant scales becomes available. In this paper, we use, without ambiguity, the following nomenclature for the four existing phases (modes): (i) inclusion phase: small cages are occupied, and large cages are empty, denoted by SOLE; (ii) matrix phase: small cages are empty and large cages are occupied; denoted by SELO; (iii) hydrogen network phase: all cages are empty, denoted by SELE; (iv) composite phase: all cages are occupied, denoted by SOLO.

This paper is organized as follows. In the results section, the inclusion and matrix phases of the sI methane hydrate were identified via polygonal connectivity through face sharing. The stability thresholds in terms of phase modes were then determined, identifying two failure patterns. The correlation between the phase occupancy and compressive stability limits is then established. The use of a ternary non-ideal laws of mixtures in conjunctions with computed DFT stability limits leads to the equation for the mechanical stability surface under variable methane occupancy. Computing the steepest descent and ascent curves leads to the identification of optimal strengthening by specific gas occupancy and maximal failure routes by a decrease of mainly large cage occupancy. All these findings can be applied to other types of gas hydrates. Finally, all methodologies and software applied in this work are presented in the SI and main text. Details of equations and mechanisms are discussed. This work contributes to and accelerates the identification of the gas hydrates’ stability under pressure. The results from this work may be crucial in developing hydrate technologies that involve the medium- and long-term storage of gases in the hydrate phase.

## Results and discussion

### Two-phase structure determination

**Figure 1:**
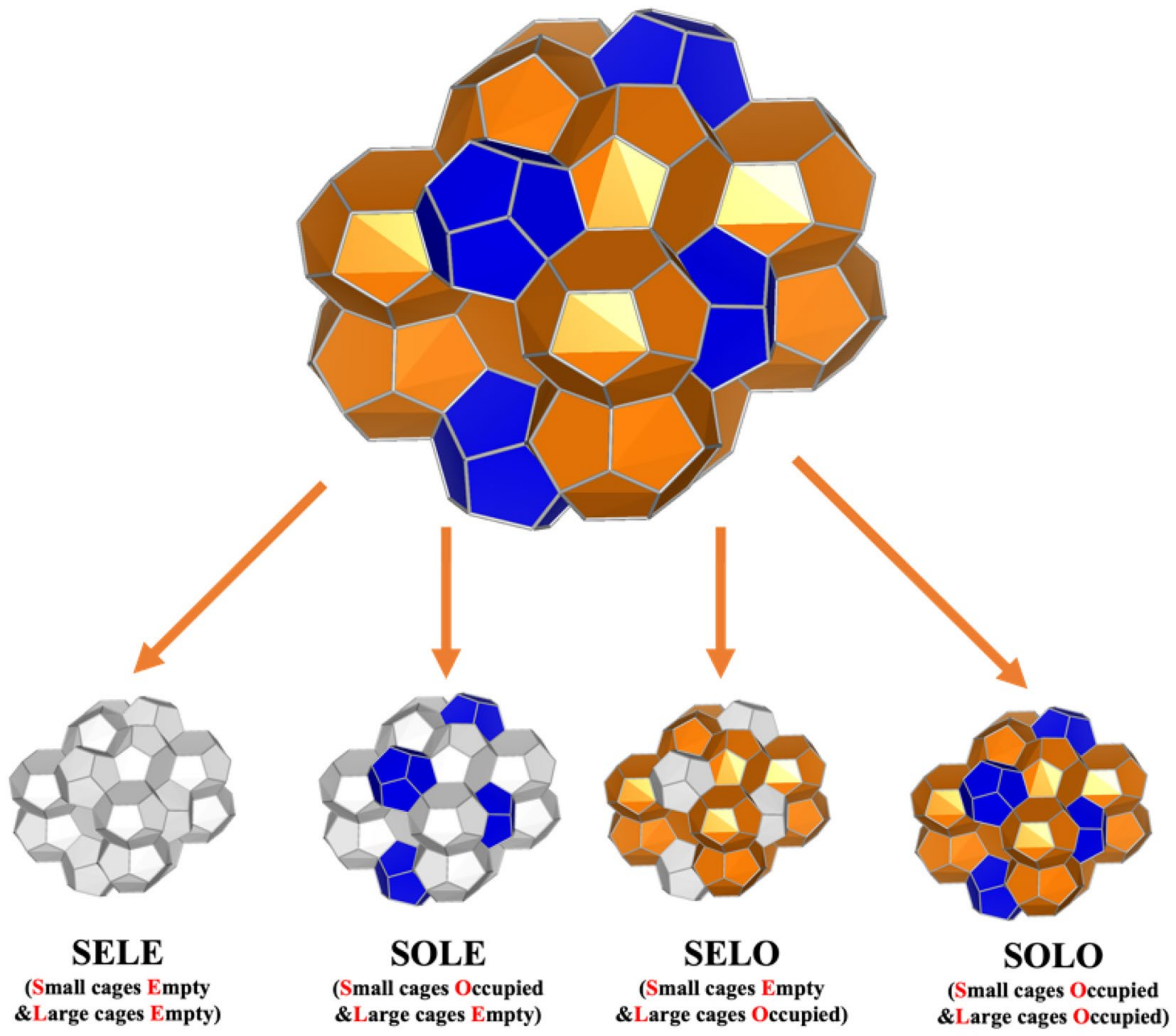
sI gas hydrate’s structure colored according to their cage types. The occupied large cages are shown in orange, the occupied small cages are shown in dark blue, and the empty cages are shown in white. Four different occupancies are shown. They are SELE (small cages empty and large cages empty), SOLE (small cages occupied and large cages empty), SELO (small cages empty and large cages occupied) and SOLO (small cages occupied and large cages occupied).

The sI gas hydrate structure consists of two small cages and six large cages. The small cages (5^12^) contain 12 pentagonal faces, and the large cages (5^12^6^2^) contain 12 pentagonal faces and two hexagonal faces. Also, taking advantage of a Three-Dimensional Visualization System for Electronic and Structural Analysis (VESTA 3)^19^, we could visualize the multi-phase structure directly, as shown in Fig. 1.

Figure 1 presents the four different occupancy modes of sI methane hydrate within one unit lattice. The occupied small cages, shown in dark blue, are distributed dispersedly. In other words, all small cages are surrounded by large cages, and they cannot connect with each other. On the other side, large cages are connected and shown in orange.

In nature, the occupancy of gas hydrates is often greater than zero, which means some gas molecules would be trapped in the lattice. However, the empty gas hydrates were confirmed to be present under certain conditions^20–23^—temperatures between 100 and 220 K and pressures between 1 and 5000 bar. Thus, four different occupancies proposed and shown at the bottom of Fig. 1 are SELE (small-empty-large-empty), SOLE (small-occupied-large-empty), SELO (small-empty-large-occupied), and SOLO (small-occupied-large-occupied). If the cages are not occupied or empty, they are shown in white. Otherwise, they are shown in their corresponding colors (dark blue for occupied small cages and orange for occupied large cages).

The continuous and dispersed roles of large and small cages in the lattice are evident in Fig. 1, which is attributed to the face connection. There are two types of faces to establish the cages and lattice: pentagonal and hexagonal faces. Pentagonal faces are involved in both small and large cages, and hexagonal faces are only formed in large cages. Each face is shared by two cages regardless of the type of cage and faces. There are six hexagonal faces in one unit lattice, and each hexagonal face would be shared by two different large cages. Also, there are 48 pentagonal faces. Half of the pentagonal faces are among large cages. However, another half is shared between one large and one small cage. According to these face-sharing principles, all faces contribute to the large cages, regardless of partial or complete involvement. In other words, if large cages exist, the small cages would be necessarily present. On the other hand, if only the small cages exist, it means that only two dispersed inclusions form since there is no shared face between the two small cages. In other words, there is no evidence to make any conclusion on large cages or lattices.

### Effects of phase occupancy on the pressure stability of hydrates

**Figure 2:**
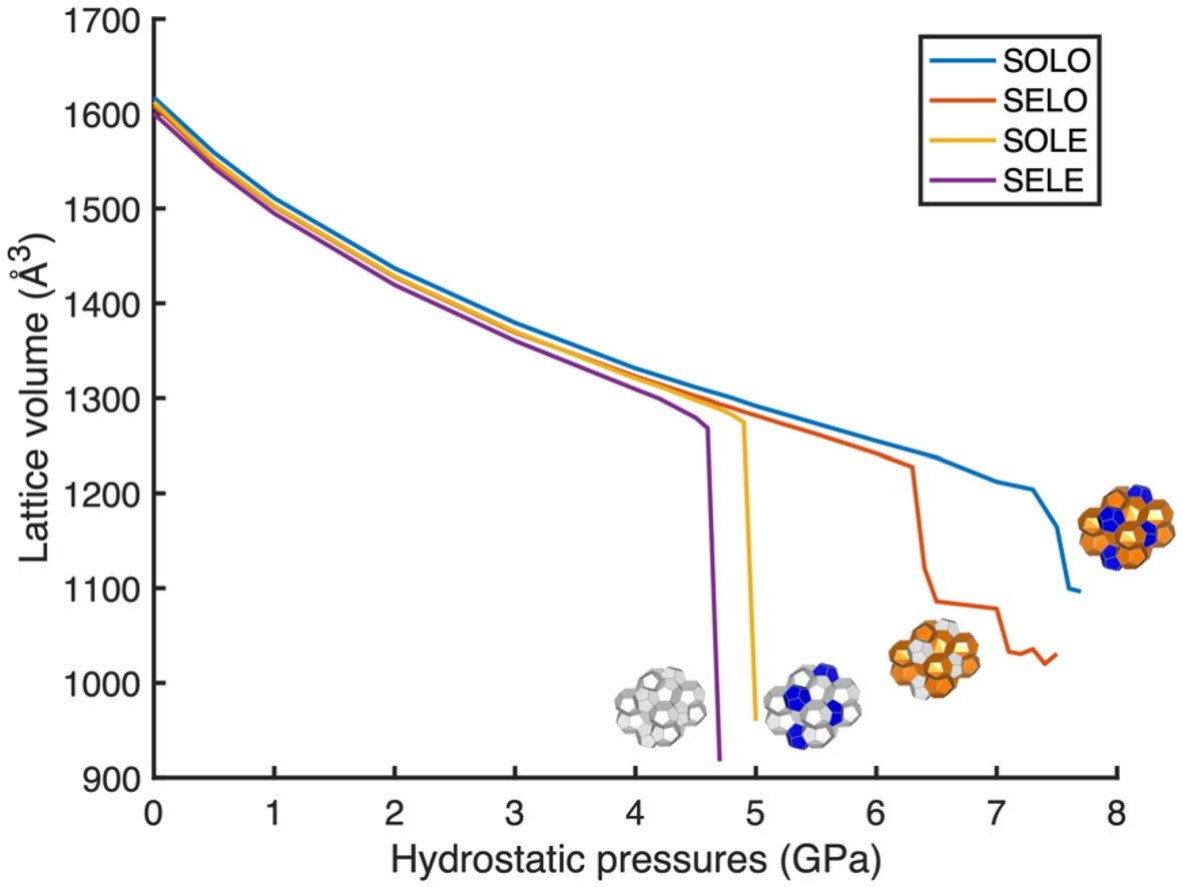
Lattice volume as a function of hydrostatic pressures from 0 to 7.6 GPa with four different occupancies. The blue line is for the SOLO hydrate. The orange line is for the SELO hydrate. The yellow and purple are for the SOLE and SELE hydrates. See text for nomenclature.

From previous study, the small cages are recognized as the dispersed inclusion and large cages are identified as the continuous phase in the lattice^16^. The simulations were performed on sI methane hydrates with four different occupancies, pressures ranging from 0 to 7.6 GPa, and at zero Kelvin; the unit lattice was the supercell. Six large tetradecahedral (5^12^6^2^) and two small dodecahedral (5^12^) polyhedral cages were involved in one unit lattice. As discussed in the previous section, four occupancies were considered: SELE, SOLE, SELO and SOLO. The effects of different occupancies (four modes mentioned above) on lattice volume under compressive pressures are shown in Fig. 2.

Figure 2 shows the variations in lattice volume under pressures ranging from 0 to 7.6 GPa. In the region of low pressure, between 0 and 4.5 GPa, the performances of all four modes are comparable. In this pressure region, all four modes exhibit a modest declining trend and a slight variation in magnitude. Compared with results from previous work^24^, the values of unit lattice volume presented here are in the same order of magnitude. For example, Jendi et al.^24^ shows the unit lattice volume of SOLO hydrate is approximately 1685 Å^3^, which is very close to our result 1620 Å^3^. At 0 GPa, the volumetric difference between SOLO occupied and SELE lattices was approximately 1%. Although SOLE has a greater lattice volume under certain low pressures than SELO, the difference is too small to determine whether occupied small cages have a stronger effect than occupied large cages. From a qualitative standpoint, we can conclude that the occupancy has no significant effect on the stability of the hydrate lattice under pressures ranging from 0 to 4.5 GPa. Based on these findings, between 0 and 4.5 GPa, the hydrogen bonding lattice supporting forces are dominant over the guest–host interaction supporting forces. This is because, under 4.5 GPa, all four cases are stable, and the volumetric difference is less than 5%. Since SELE hydrate failed at 4.7 GPa, 4.6 GPa was considered its compressive stability limit. The cage volumes for empty sI hydrate under 4.6 GPa are also considered the minimum hydrogen-bonded tetradecahedral (5^12^6^2^) and dodecahedral (5^12^) cages volumes, respectively, as shown in Table 1.

**Table 1:** Cage sizes for empty sI hydrate under 4.6 GPa, where the empty hydrate can support the maximum compressive pressure.

Small cages	Cage 1			Cage 2		
Cage size (Å^3^)	123.94			128.87		
Large cages	Cage 3	Cage 4	Cage 5	Cage 6	Cage 7	Cage 8
Cage size (Å^3^)	181.55	181.55	171.68	171.68	171.29	171.29

Cages 1 and 2 are the small cages, and cages 3–8 are the large cages.

Table 1 shows detailed information on cage sizes for empty sI hydrate under 4.6 GPa. Small cages have volumes around 120 Å^3^, the large cages would have volumes ranging from 171 to 182 Å^3^. Since these values represent the cage sizes under maximum pressure without losing lattice integrity, these numbers can serve as minimum stable volumes for tetradecahedral (large) and dodecahedral (small) cages. In other words, any smaller size without additional supporting force (e.g., the presence of guest molecules) can be identified as cage failure.

Figure 2 shows that once the pressure exceeds 4.5 GPa, hydrates having different occupancies respond differently. This is because the guest–host interaction starts to become a dominant effect. The figure allows for the classification of the four modes into two groups. The SOLO and SELO cases had comparable trends, while the SOLE and SELE cases exhibited similar behavior. The volume sizes of SOLO and SELO hydrates overlap when the pressure is below 6.4 GPa. However, once the pressure increased from 6.3 to 6.4 GPa, the lattice volume of the SELO case unexpectedly decreased from 1227.72 to 1121.34 Å^3^, where the volume changes are ten times greater than the amount of volume change under a lower pressure with the same magnitude of pressure change. A similar volume drop takes place on the SOLO hydrates but under greater pressure (7.3 GPa). After the first drop, both SELO and SOLO hydrates seem to reach a plateau. After the plateau, the SELO and SOLO hydrates had their second volume drop at 7.1 GPa and 7.6 GPa, respectively, followed by a short plateau again. In Fig. 2, SELO shows more discrete changes in lattice volume than SOLO because SELO has a 1.3 GPa pressure range to show its failure response while SOLO only has 0.3 GPa. This pressure range is determined between the maximum simulation pressure limits 7.6 GPa and their first volume drop pressure. In other words, the SOLO structure exhibited a higher resistance to volume change (higher mechanical stability) than the SELO structure, due to the occupation of its small cages.

However, the SOLE and SELE hydrates have completely different responses. The SOLE and SELE hydrates have their first drop under 4.9 GPa and 4.6 GPa respectively. Unlike the SOLO and SELO hydrates, no plateau emerges after the volume drop, and it seems they shrink to their minimum volume, where no lattice structure exists. In other words, the volume size represents the sum of the atoms and molecules in the lattice. Comparing to previous work^15,16^, the compressive pressure limit for SOLO sI methane hydrate is 7.5 GPa, which is in agreement with the first volume drop of SOLO hydrate at 7.3 GPa shown in Fig. 2. The results’ reliability and accuracy are further discussed below.

### Relationship between phase occupancy and compressive stability limits

In the previous section we obtained the maximum pressures, which were recognized as the stability limits, that the hydrate structure can withstand without sacrificing the lattice volume. Considering the law of mixtures, the property of the composite material is the weighted average of the property values of constituent materials. This idea can be extended to the prediction of stability limits, where the volumetric ratio is replaced by the lattice occupancy with respect to small and large cages.

The differences among first volume drop critical pressure points imply a relationship between the phase occupancy and compressive stability limits. The effects from occupied small and large cages are significantly different, and the interactions among occupied cages are important. Thus, in this section, we will quantify the impacts of these effects under pressure.

Table 2, shows the critical pressure points for the first significant volume drop for sI methane hydrates with four different occupancies (see Fig. 2). In general, the pressure at which the first volume drop occurs increases as the occupancy increases, which is reasonable. Since more cages are occupied, more guest–host interactions can be used to stabilize the lattice. For quantification, two assumptions were made to establish an equation between the critical pressure points (pressures for the first significant volume drop) and phase occupancy. Firstly, it is linearly correlated between the phase occupancy and critical pressure points. Secondly, only the interactions among different cages are considered. The interactions within the same type of cages are ignored.

**Table 2:** Compressive pressure points (see Fig. 2) for first significant volume drop for sI methane hydrates with four different occupancies (SELE, SOLE, SELO and SOLO).

SELE	SOLE	SELO	SOLO
4.6 GPa	4.9 GPa	6.3 GPa	7.3 GPa

Four modes and a canonical equation are proposed to quantify the occupancy effects on the compressive stability limits, as shown in Eq. (1). Although Eq. (1) is inspired from the ideal law of binary mixtures, they have four main differences. Firstly, there are three components in Eq. (1) (hydrogen-bonded skeleton, small and large cage’s occupancies) instead of two in law of mixtures. Secondly, the variables X and Y in Eq. (1) have values between 0 and 1; however, in law of binary mixtures the sum of the volumetric ratio is constrained to 1. Thirdly, in Eq. (1), the (X,Y) variables are occupancy instead of the volumetric ratio. Lastly, cage occupancy interaction between large and small leads naturally to the last term in Eq. (1). The analysis and DFT data is synthesized in terms of a stability surface S(X,Y):

1 $${\text{S }}\left( {X,Y} \right) = {\text{a}} + {\text{bX}} + {\text{cY}} + {\text{dXY}}$$

where S stands for the compressive stability limit (GPa), X is the small cages’ occupancy and Y is the large cages’ occupancy, and a, b, c, and d are constants. There are four terms in Eq. (1). One constant term shows as ‘a’. Two linear correlated terms, ‘bX’ and ‘cY’, are used to describe the occupied small and large cages, respectively. The fourth term ‘dXY’ describes the interaction between occupied small and large cages. According to the four different occupancies mentioned above and, in the introduction, four modes are proposed, as shown Table 3. The network mode, which represents the hydrogen-bonded skeleton, describes the SELE hydrate. The inclusion and matrix modes demonstrate the SOLE and SELO hydrates. The composite mode describes the SOLO hydrates.

**Table 3:** The four occupancy modes that contribute to the overall compressive stability model in Eq. (1).

Network mode (SELE)	${\text{S}} = {\text{a}}$
Inclusion mode (SOLE)	${\text{S}} = {\text{a}} + {\text{bX}}$
Matrix mode (SELO)	${\text{S}} = {\text{a}} + {\text{cY}}$
Composite mode (SOLO)	${\text{S}} = {\text{a}} + {\text{bX}} + {\text{cY}} + {\text{dXY}}$

With the four critical points shown in Table 2, the assumption listed above and the proposed generic surface stability equation, we obtained a specific equation, as shown in Eq. (2). The constant term, 4.6, is from the SELE (empty) hydrates. The coefficients 0.3 and 1.7 are obtained from the differences between SOLE and SELE and between SELO and SELE, where the extended stability limits are only from the occupied small or large cages. To estimate the interactions between different types of cages, we calculated the differences between SOLO and SOLE and between SOLO and SELO. Both are 0.7 GPa, indicating that large and small cages would boost another type of occupied cages by 0.7 GPa. In Eq. (2), S stands for the compressive critical pressure points or stability limits. X and Y are replaced by Q_s_ and Q_l_, which are the small and large cage occupancy respectively (between 0 and 1).

2 $${\text{S }}\left( {{\text{GPa}}} \right) = 4.6 + 0.3 \times {\text{Q}}_{{\text{s}}} + 1.7 \times {\text{Q}}_{{\text{l}}} + 0.7 \times {\text{Q}}_{{\text{s}}} {\text{Q}}_{{\text{l}}}$$

The detailed comparisons between ideal constrained binary and nonlinear unconstrained ternary mixture models are shown in the Supporting Information (SI1).

**Figure 3:**
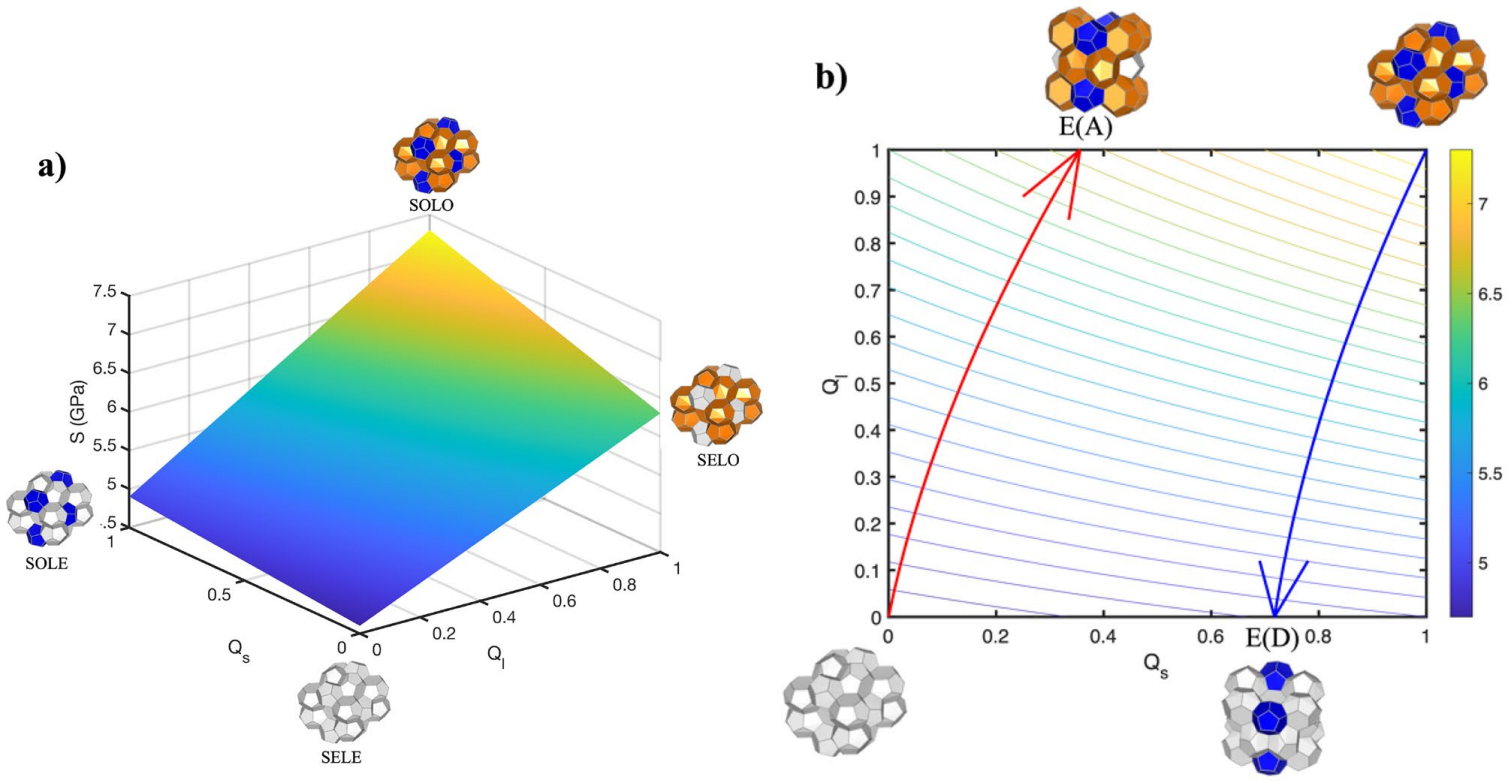
Relationship between compressive stability limit and phase occupancy. Q_s_ and Q_l_ are the occupancies of small and large cages, respectively. (**a**) A 3D surface shows the compressive stability limits change with phase occupancy. (**b**) indicates the contour level with step size 0.1 GPa. The red arrow curve is the steepest ascent curve, and the blue arrow curve is the steepest descent curve, which is the fastest way to increase and decrease stability limits. E(A) and E(D) indicate the ending points for the ascent and descent curves, respectively. The color bar represents the stability limit values from 4.6 to 7.3 GPa, with a unit of GPa.

A 3D surface and contour levels were generated for Eq. (3), as shown in Fig. 3. Figure 3a shows the relationship between the compressive stability limits and phase occupancy (Q_s_ and Q_l_). The stability limits range from 4.6 to 7.3 GPa. Figure 3b shows the contour levels with a step size of 0.1 GPa.

The two curves in Fig. 3b are generated by using the standard steepest ascent/descent method. The red curve shows the steepest changing pathway to increase the stability limits from empty hydrates, and the blue curve shows the steepest changing pathway to decrease the stability limits from fully occupied hydrates. The steepest ascent curve ends at $E\left( A \right) = \left( {0.3524,1} \right)$,where the stability limit equals to 6.65 GPa. The steepest descent curve ends at $E\left( D \right) = \left( {0.7136,0} \right)$, where the stability limit equals to 4.81 GPa. From the values of ending points, we find that the values of Q_l_ are either 1 or 0, which means the occupancy of large cages has the dominant role for the entire lattice’s stability. On the other hand, the difference between the Q_s_ values of E(A) and of (D) is relatively small, indicating the secondary role of small cage occupancy.

Using the steepest descent/ascent curves of the stability surface, predictions to capture the relationship between cage occupancy to achieve the fastest way to increase or decrease the stability limits can be established as follows:

3 $$0.35\left( {Q_{l}^{2} - Q_{s}^{2} } \right) + 0.3\left( {Q_{l} - 5.67Q_{s} } \right) = K_{i} \left( {GPa} \right), i = A,D$$

$i = A, K_{A} = 0$: steepest ascent from SELE (network) state, $i = D, K_{D} = - 1.4:$ steepest descent from SOLO (composite) state.

Equation 3 (see SI for derivation) shows that the relationship between unconstrained occupancy $Q_{l}$ and $Q_{s}$ is hyperbolic, reflecting the impact difference between large and small cage effects. The stability limits’ values for the terminal stage for optimal strengthening E(A) and for largest stability drop E(D) are:

4 $$S\left( {E\left( A \right)} \right) = S_{max} - \left( {b + d} \right)\left[ {1 + \frac{{c - \sqrt {c^{2} + 2bd + d^{2} } }}{d}} \right]$$

5 $$S\left( {E\left( D \right)} \right) = S_{min} + \frac{{ - c + \sqrt {c^{2} - 2bd + 2dc} }}{d}$$

where $\left\{ {a, b, c, d} \right\} = \left\{ {4.6, 0.3, 1.7, 0.7} \right\}$ (coefficients in Eq. (2)); $S_{max} = S\left( {Q_{s} = 1,Q_{l} = 1} \right)$ and $S_{max} = a + b + c + d; S_{min} = S\left( {Q_{s} = 0,Q_{l} = 0} \right) = a.$ Equations (4, 5) explain why the end points (E(A), E(D) in Fig. 3) do not achieve the minimum and maximums stability limits values when following the dominant curves of steepest ascent/descent curves of the stability surface S. The presence of second terms in Eqs. (4, 5), accounting for cages (coefficients b and c) and cage interaction (coefficient d) effects, indicate the difference. This mechanical response to composition clearly indicates the limitations of the optimal path to achieve maximal strength and the destabilizing nature of losing large cage occupancy.

To evaluate the consistency between the DFT stability limits and the values calculated by using the mixture model (Eq. (2)), three arbitrary occupancy modes were selected. Detailed information on occupancy is shown in Table 4.

**Table 4:** Comparison between DFT simulations and mixture model (Eq. (2)) with 50% occupancy in three representative modes.

	Inclusion mode $Q_{s} = 0.5; Q_{l} = 0$	Matrix mode $Q_{s} = 0; Q_{l} = 0.5$	Composite mode $Q_{s} = 0.5; Q_{l} = 0.5$
DFT Stability limits (GPa)	4.7	5.2	5.6
Calculated stability limits by using Eq. (2) (GPa)	4.75	5.45	5.95

Table 4 shows the compressive stability limits for three representative cases; the inclusion mode has only 50% of small cages being occupied, the matrix mode has only 50% of large cages occupied, and the composite mode has 50% of small cages and 50% of large cages occupied. These three modes with 50% occupancy provide a clear understanding of both individual cages’ effects and their interactions. DFT simulations for the three modes have precisions of 0.1 GPa, and the precision cannot be smaller due to computational cost. The DFT results are obtained by relaxing the three selected modes of hydrates under pressure and finding the maximum pressure that the hydrate lattice can support before first significant volume drop. Table 4 shows that the DFT simulation and the mixture model (Eq. (2)) results are in very or excellent agreement. Although values from Eq. (2) can be greater than the simulation results, the discrepancies are always less than 10%, which implies the validity of the previously mentioned two assumptions and further confirms the accuracy of Eq. (2).

### Failure mechanisms

Figure 2 shows two different behaviors when the system approaches instability thresholds. The SELE and SOLE hydrates show a sudden step-like volumetric drop. On the other hand, the SOLO and SELO hydrates showed sequential staircase-like volume drops. To further understand the reasons for the sequential volume drops, we use Visualization for Electronic Structural Analysis (VESTA), to ascertain if there is any failure or intermolecular distortion in the lattice.

**Figure 4:**
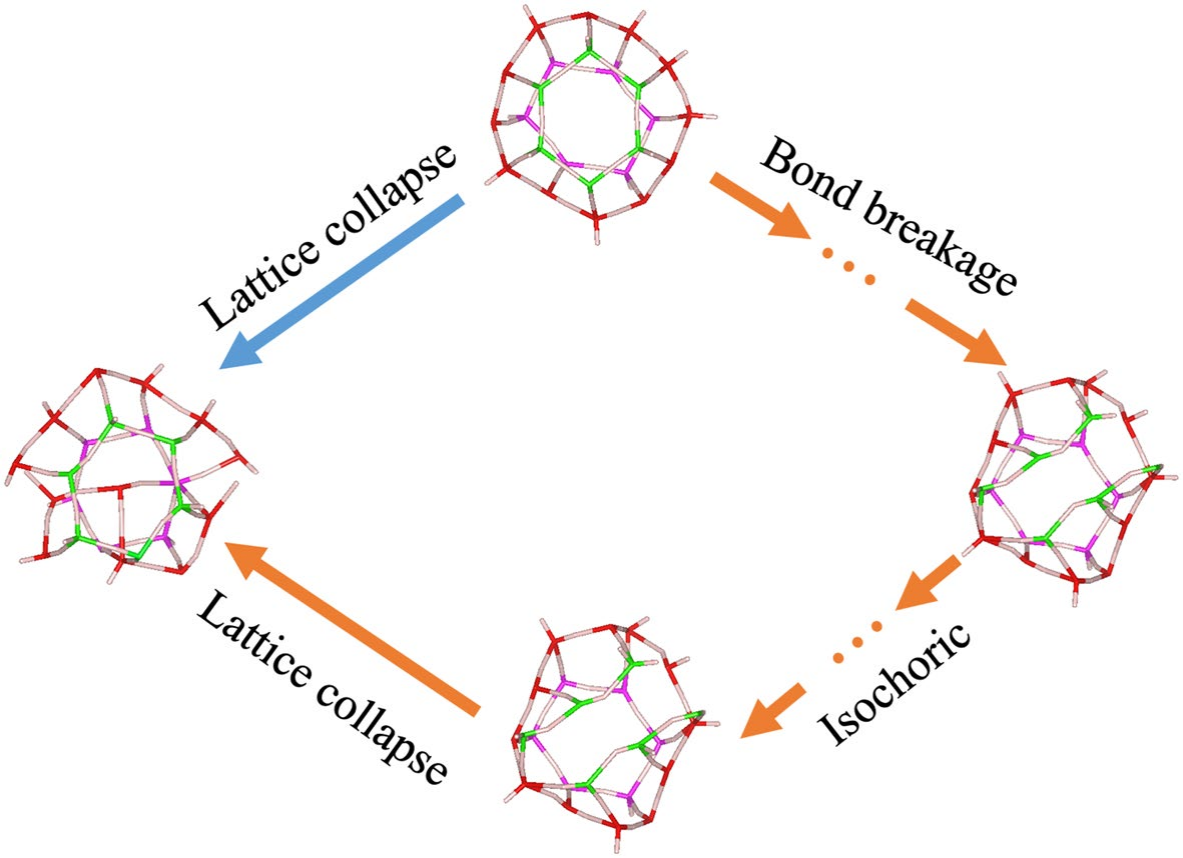
Two failure mechanisms when approaching the compressive stability limit. The Failure Mechanism 1 (orange arrowed lines) found in SOLO and SELO hydrates has three steps: bond breakage, isochoric deformation, and lattice collapse. The arrow-dot-arrows represent sequential steps that take places several times before the lattice fails. The bond breakage step shows a significant volume drop, where bonds sacrifice at high pressures. The isochoric step indicates the hydrate system reaches a plateau with no volume change even when additional pressures are applied. Lattice collapse is the last step and represents a systematic failure. The Failure Mechanism 2 found in SELE, and SOLE hydrates is shown in blue. It has only one step and is the same as the last step of Failure Mechanism 1, where the system lost its lattice integrity.

Figure 4 shows the two failure mechanisms that take place when the compressive stability limits are approached. The Failure Mechanism 1 (orange arrows) has three steps and Failure Mechanism 2 has only one step (blue arrows). Both mechanisms involve significant volume drops. The Failure Mechanism 1 was shown in the SOLO (composite) and SELO (matrix) hydrates. Meanwhile, the Failure Mechanism 2 was shown in the SELE (network) and SOLE (inclusion) hydrates.

The existence of two failure mechanisms is determined by how widely distributed the supporting forces are. Above 4.5 GPa, the hydrogen bond system cannot support the cage and lattice by itself. Thus, the guest–host interaction becomes very important for the hydrate’s stability. Both SELE (network) and SOLE (inclusion) hydrates display the Failure Mechanism 2 because they do not have any occupied large cages. As discussed above, the small cages are dispersed and do not connect with each other. Thus, guest–host interactions can only be limited to local areas. As expected, the SOLE hydrates do have a greater stability limit than SELE, as shown in Fig. 2, but the same failure pathway.

However, the SELO (matrix) and SOLO (composite) hydrates exhibit Failure Mechanism 1 instead of Failure Mechanism 2. Failure Mechanism 1 is a sequential or multi-step mechanism. At sufficient low pressure, although the cages are deformed, the faces’ and cages’ integrity were present. However, once the pressure reaches the first volume drop threshold, one hexagonal ring opens by bond breakage. It is possible that a couple of pentagonal faces sacrifice instead of hexagonal faces, but the main idea is that the cage integrity remains. At the same time, some bonds break, causing a significant volume drop. The two layers of pentagonal rings are the main body of the large cages. If they are complete and intact, the lattice’s guest-hosting capability is still present. In the isochoric zone, the change in volume is negligible compared to the magnitude of the total volume. This zone does experience a small volume decrease, as seen in Fig. 2. From Fig. 2, both SELO (matrix) and SOLO (composite) hydrates show that the lattice volume becomes constant for a while after a significant volume drop, even when additional pressure is applied. This can be explained as the structure has changed to a new stable structure under pressure. Bond breakage and isochoric steps may take place several times before the final step (lattice collapse) is triggered. In summary, the volume drops and stabilizes several times before the final collapse. Due to computational costs and capabilities, the results beyond 7.6 GPa are out of convergence reach, and the final collapses of SOLO and SELO hydrates are not captured. But with all the information we have so far, the failure mechanism can be deduced without any ambiguities.

## Methods and tools

### DFT simulations and computational analyses

First principle DFT calculations were performed by using VASP software. To obtain the lowest energy system under different pressures, we must relax the hydrate lattice in all three degrees of freedom (e.g., atomic positions, lattice volume and structural shape). The sI methane hydrates consist of the water molecules forming the host cages and methane molecules are the guest molecules. For the water cages, the water oxygen atoms’ positions can be determined by X-ray diffraction, with the positions of water hydrogen atoms proposed by Takeuchi et al.^25^, which followed the Bernal-Fowler ice rules^26^. Methane molecules were selected to be the only guest molecules in the simulation box. They were placed at the center of each cage in the initial structure.

**Figure 5:**
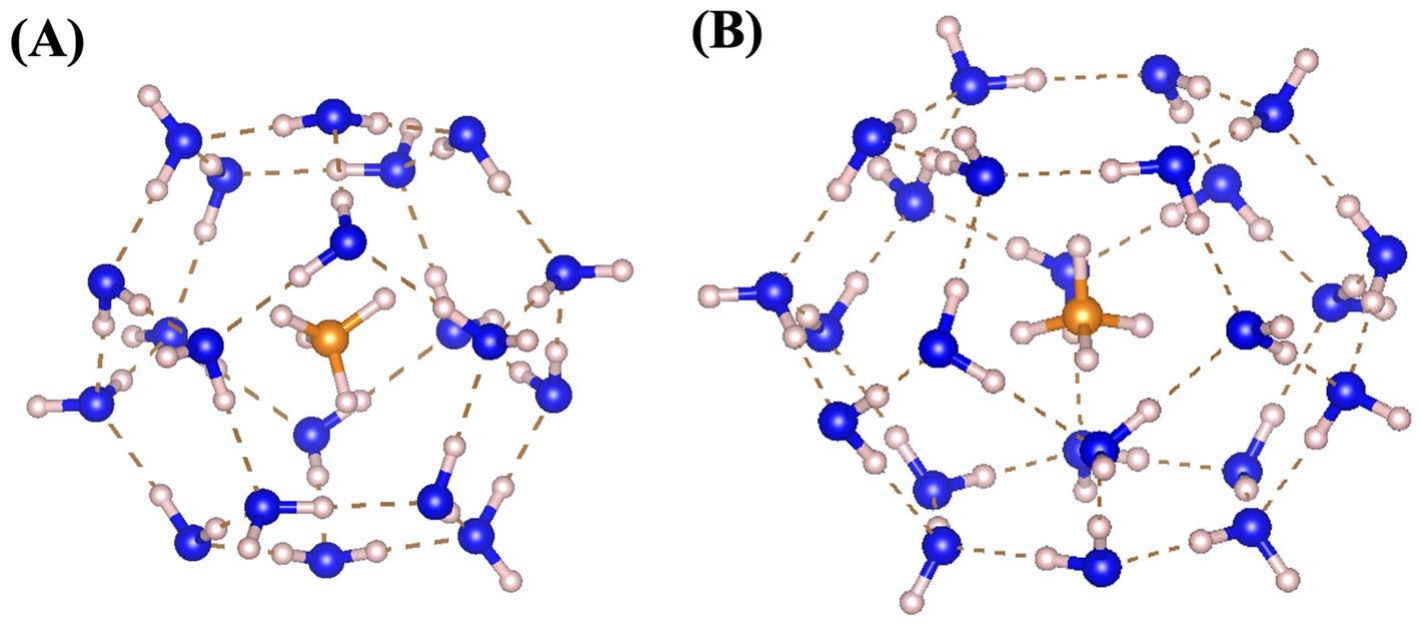
sI methane hydrates have small (**A**) and large (**B**) cages. (**A**) A small cage consists of 12 pentagonal faces and a methane molecule at its center. Large cages (**B**) have 12 pentagonal faces and two hexagonal faces, with a guest methane molecule at the center. Water oxygen atoms are represented by blue circles. Hydrogen atoms are represented by pink-white circles. Carbon atoms are denoted by orange circles. All solid lines represent covalent bonds; their colors indicate which two atoms formed the covalent bond. OH hydrogen bonds are displayed by the brown dashed lines.

In the unit lattice of sI hydrate there are 46 water molecules, which form two small cages and six large cages. The small cages (5^12^) contain 12 pentagonal faces and the large cages (5^12^6^2^) contains 12 pentagonal faces and two hexagonal faces. Considering the size of host cages and guest molecules, each cage can only have one methane molecule, regardless the cage type. The details of small and large cages are shown in Fig. 5.

As mentioned above, VASP was selected as the DFT implementation for all simulations in this work. The simulation involves one unit lattice of sI methane hydrate shown above at zero Kelvin with periodic boundary conditions. The conjugate-gradient algorithm was used since it has a reliable performance within or beyond the pressure stability limits^15^. Based on previous studies on gas hydrate^2,5,8,15,16^, the revised Perdew-Burke-Ernzerhof (revPBE) exchange–correlation functional and the DFT-D2 dispersion correction method was selected^15,16,27^. Projector augmented wave potential^28,29^ was employed for all calculations. Furthermore, 520 eV was chosen as the cut-off energy for the entire simulation process. As recommended by the VASP manual^30^, the cut-off energy needs to be set at a level that is 30% higher than the maximum energy of all the atoms that were included in the simulation. For the electronic minimization, a 4 $\times$ 4 $\times$ 4 Gamma centered mesh was used^15,16^. Four subdivisions were selected in each reciprocal lattice vector considering the results’ accuracy and computational cost. All simulation details can be found in our previous work^15,16^, including the unit lattice preparation, parameter setting and structure relaxation.

### Contour levels and steepest decent method

The contour levels and steepest decent method are applied for the surface generated by Eq. (4). The contour levels are set with the step size of 0.1 GPa to show how values of stability limits evolve with occupancy. The steepest descent method was used to generate two critical curves to show the fastest pathways to increase or decrease the stability limits. The algorithm is shown below. $F\left( {X,Y} \right)$ is a multi-variable function with variables X and Y. The initial points are selected as $x_{0}$ and $y_{0}$. $\gamma$ is the step size. In this paper, a fixed step size of 0.02 was selected. The values are small enough to capture all the changing tendencies. The optimization method of step size was not applied, because we have some restrictions in this project. For example, the stability limits cannot be negative. Thus, a fixed step size with a small enough value would be the best choice. Calculations would keep iterated until ${\text{F}}\left( {{\text{x}}_{{{\text{n}} + 1}} ,{\text{y}}_{{{\text{n}} + 1}} } \right) < {\text{Lower Limit }}$ or ${\text{F}}\left( {{\text{x}}_{{{\text{n}} + 1}} ,{\text{y}}_{{{\text{n}} + 1}} } \right) > {\text{Upper Limit}}$.

6 $$x_{n + 1} = x_{n} \pm \gamma \nabla F\left( {x_{n} } \right)$$

7 $$y_{n + 1} = y_{n} \pm \gamma \nabla F\left( {y_{n} } \right)$$

Alternatively, to this standard numerical approach, the equations in SI 2 can be used to calculate the steepest ascent/descent curves.

## Supplementary Information

Supplementary Information.

## Data Availability

Data related to this work will be made available by request to the authors. These requests should be addressed to the corresponding author A.D.R. at alejandro.rey@mcgill.ca.

## References

[1] Sloan ED, Koh CA, Koh CA (2007). Clathrate Hydrates of Natural Gases.

[2] Vlasic TM, Servio P, Rey AD (2016). Atomistic modeling of structure II gas hydrate mechanics: Compressibility and equations of state. AIP Adv..

[3] Daghash SM, Servio P, Rey AD (2019). Structural properties of sH hydrate: A DFT study of anisotropy and equation of state. Mol. Simul..

[4] Daghash SM, Servio P, Rey AD (2020). From infrared spectra to macroscopic mechanical properties of sH gas hydrates through atomistic calculations. Molecules.

[5] Daghash SM, Servio P, Rey AD (2021). First-principles elastic and anisotropic characteristics of structure-H gas hydrate under pressure. Crystals.

[6] Vlasic TM, Servio PD, Rey AD (2019). Infrared spectra of gas hydrates from first-principles. J. Phys. Chem. B.

[7] Mathews SL, Servio PD, Rey AD (2020). Heat capacity, thermal expansion coefficient, and Grüneisen parameter of CH_4_, CO_2_, and C_2_H_6_ hydrates and Ice Ih via density functional theory and phonon calculations. Cryst. Growth Des..

[8] Jendi ZM, Servio P, Rey AD (2015). Ideal strength of methane hydrate and Ice Ih from first-principles. Cryst. Growth Des..

[9] Jia J, Liang Y, Tsuji T, Murata S, Matsuoka T (2017). Elasticity and stability of clathrate hydrate: Role of guest molecule motions. Sci. Rep..

[10] Mirzaeifard S, Servio P, Rey AD (2018). Molecular dynamics characterization of temperature and pressure effects on the water-methane interface. Colloid Interface Sci. Commun..

[11] Guerra A (2022). Dynamic viscosity of methane and carbon dioxide hydrate systems from pure water at high-pressure driving forces. Chem. Eng. Sci..

[12] Guerra A, Mathews S, Marić M, Servio P, Rey AD (2022). All-atom molecular dynamics of pure water–methane gas hydrate systems under pre-nucleation conditions: A direct comparison between experiments and simulations of transport properties for the Tip4p/Ice water model. Molecules.

[13] Guerra, A. *et al.* Molecular dynamics predictions of transport properties for carbon dioxide hydrates under pre-nucleation conditions using TIP4P/Ice water and EPM2, TraPPE, and Zhang carbon dioxide potentials. Preprint https://arxiv.org/abs/2301.01757 (2023).

[14] Wu J (2015). Mechanical instability of monocrystalline and polycrystalline methane hydrates. Nat. Commun..

[15] Zhu X, Rey AD, Servio P (2022). Piezo-elasticity and stability limits of monocrystal methane gas hydrates: Atomistic-continuum characterization. Can. J. Chem. Eng. n/a.

[16] Zhu X, Rey AD, Servio P (2022). Multiscale piezoelasticity of methane gas hydrates: From bonds to cages to lattices. Energy Fuels.

[17] Deserno, M. *Notes on Differential Geometry with Special Emphasis on Surfaces in R3* (2004).

[18] Guerra A, Mathews S, Marić M, Rey AD, Servio P (2022). An integrated experimental and computational platform to explore gas hydrate promotion, inhibition, rheology, and mechanical properties at McGill University: A review. Energies.

[19] Momma K, Izumi F (2011). VESTA3 for three-dimensional visualization of crystal, volumetric and morphology data. J. Appl. Crystallogr..

[20] Cruz FJAL, Alavi S, Mota JPB (2019). Low-temperature thermodynamic study of the metastable empty clathrate hydrates using molecular simulations. ACS Earth Space Chem..

[21] Falenty A, Hansen TC, Kuhs WF (2014). Formation and properties of ice XVI obtained by emptying a type sII clathrate hydrate. Nature.

[22] Kosyakov VI (2009). Structure formation under negative pressures. J. Struct. Chem..

[23] Conde MM, Vega C, Tribello GA, Slater B (2009). The phase diagram of water at negative pressures: Virtual ices. J. Chem. Phys..

[24] Jendi ZM, Rey AD, Servio P (2015). Ab initio DFT study of structural and mechanical properties of methane and carbon dioxide hydrates. Mol. Simul..

[25] Takeuchi F (2013). Water proton configurations in structures I, II, and H clathrate hydrate unit cells. J. Chem. Phys..

[26] Bernal JD, Fowler RH (1933). A theory of water and ionic solution, with particular reference to hydrogen and hydroxyl ions. J. Chem. Phys..

[27] Jendi ZM, Rey AD, Servio P (2015). Ab initioDFT study of structural and mechanical properties of methane and carbon dioxide hydrates. Mol. Simul..

[28] Blöchl PE (1994). Projector augmented-wave method. Phys. Rev. B.

[29] Kresse G, Joubert D (1999). From ultrasoft pseudopotentials to the projector augmented-wave method. Phys. Rev. B.

[30] Georg Kresse, M. M., Jurgen Furthmuller. (2018).

