# Supplementary material for: Atomistic-geometric simulations to investigate the mechanical stability of monocrystalline sI methane hydrates under pressure
Source: Sci Rep. 2023 Feb 2;13:1907. doi: 10.1038/s41598-023-29194-8 (PMC9894853; doi:10.1038/s41598-023-29194-8)
Supplement: Supplementary file 1 — Supplementary Information. [file 41598_2023_29194_MOESM1_ESM.docx]

**Atomistic-geometric simulations to investigate the mechanical stability of monocrystalline sI methane hydrates under pressure**

Authors: Xiaodan Zhu^1^, André Guerra^1^, Phillip Servio^1^, Alejandro D. Rey^1^*

^1^Department of Chemical Engineering, McGill University, Montreal, Quebec, Canada, H3A 0C5

***Correspondence**: Alejandro D. Rey, McGill University, [alejandro.rey@mcgill.ca](mailto:alejandro.rey@mcgill.ca)

**SI 1: Binary and Ternary Models**

Binary (two-component mixture) and Ternary (three-component mixture) were proposed in our previous and current work. This section aims to compare binary mixture model at full occupancy with a corresponding ternary mixture mode with no composition constraint.

Nomenclature:

**Binary Model:** Two-component mixture model at full occupancy. This model is only valid at full occupancy because partial occupancy implies free volume and in a real material the hydrogen network spans the system

Physical Property: P^2^

$$S:small cage$$

$$L:large cage$$

$$N_{S/L}: number of small or large occupied cages in one unit lattice$$

$$V:total volume of one unit lattice$$

$$v_{S/L}: volume of a single small or large cage$$

$$V_{S/L}: volume of small or large occupied cages in one unit lattice$$

$$f:a required condition of at full occupancy$$

$$\varphi_{S/L}: volume fraction of occupied small or large cage in one unit lattice$$

$$P^{2S/2L}:the property of a single occupied small or large cage$$

$$\varphi_{Sf}=\frac{total volume of all small cages}{total lattice volume}=\frac{N_{sf}v_{s}}{V}=\frac{V_{sf}}{V}=\alpha$$

$$\varphi_{Lf}=\frac{total volume of all large cages}{total lattice volume}=\frac{N_{Lf}v_{L}}{V}=\frac{V_{Lf}}{V}=\beta$$

Constraint: $\varphi_{Sf}+\varphi_{Lf}=\alpha+\beta=1$

Property:

| $P^{2}=\varphi_{Sf}P^{2S}+\varphi_{Lf}P^{2L}=\alpha P^{2S}+\beta P^{2L}$ | Eqn (S1) |
| --- | --- |

**Ternary Model:** Three-component mixture model with bounded occupancy constraint

Physical Property : P^3^

$$HN:hydrogen-bonded network$$

$$S:small cage$$

$$L:large cage$$

$$o_{S}:occupany of small cages$$

$$o_{L}:occupancy of large cages$$

$$Bounds:0\leq o_{S}\leq1; 0\leq o_{L}\leq1$$

$$Bounded constraint: 0\leq o_{S}+o_{L}\leq2$$

$$P^{{3S}/{3L}}:property of occupied small or large cages$$

$$P^{3HN}:property of hydrogen-bonded network$$

$$P^{3I}:property of interaction between occupied small and large cages$$

$$o_{S}=\frac{number of occupied small cages in one unit lattice}{total number of small cages in one unit lattice}=\frac{N_{s}}{N_{sf}}$$

$$o_{L}=\frac{number of occupied large cages in one unit lattice}{total number of large cages in one unit lattice}=\frac{N_{L}}{N_{Lf}}$$

| $\varphi_{S}=\frac{N_{s}v_{s}}{V}$ | Eqn (S2) |
| --- | --- |

| $\varphi_{L}=\frac{N_{L}v_{L}}{V}$ | Eqn (S3) |
| --- | --- |

| $\frac{o_{s}}{V}=\frac{N_{s}}{N_{sf}V}\times\frac{v_{s}}{v_{s}}=\frac{\varphi_{S}}{N_{sf}v_{s}}$ | Eqn (S4) |
| --- | --- |

| $\frac{o_{L}}{V}=\frac{N_{L}}{N_{Lf}V}\times\frac{v_{L}}{v_{L}}=\frac{\varphi_{L}}{N_{Lf}v_{L}}$ | Eqn (S5) |
| --- | --- |

| $o_{s}=\frac{V}{N_{sf}v_{s}}\times\varphi_{S}=\frac{\varphi_{S}}{\varphi_{Sf}}$ | Eqn (S6) |
| --- | --- |

| $o_{L}=\frac{V}{N_{Lf}v_{L}}\times\varphi_{L}=\frac{\varphi_{L}}{\varphi_{Lf}}$ | Eqn (S7) |
| --- | --- |

**Model P^3^ at arbitrary occupancy in terms of volume fractions:**

**NOTE:** $\boldsymbol{\varphi}_{\boldsymbol{L}}\boldsymbol{+}\boldsymbol{\varphi}_{\boldsymbol{S}}\boldsymbol{\neq1}$

Property:

| $P^{3}=P^{3HN}+o_{S}P^{3S}+o_{L}P^{3L}+{o_{S}o}_{L}P^{3I}$ | Eqn (S8) |
| --- | --- |

Based on Eqn (S6) and Eqn (S7), Eqn (S8) can be shown as Eqn (S9)

| $P^{3}=P^{3HN}+\frac{\varphi_{S}}{\varphi_{Sf}}P^{3S}+\frac{\varphi_{L}}{\varphi_{Lf}}P^{3L}+\frac{\varphi_{S}}{\varphi_{Sf}}\frac{\varphi_{L}}{\varphi_{Lf}}P^{3I}$ | Eqn (S9) |
| --- | --- |

**Mapping ternary to binary at full occupancy**

**At full occupancy**

$$\left\{ \begin{aligned} \varphi_{L}=\varphi_{Lf} \\ \varphi_{S}=\varphi_{Sf} \end{aligned} \right.$$

$$\varphi_{L}+\varphi_{S}=\varphi_{Lf}+\varphi_{Sf}=1$$

$$o_{s}=\frac{\varphi_{S}}{\varphi_{Sf}}=1$$

$$o_{L}=\frac{\varphi_{L}}{\varphi_{Lf}}=1$$

$$o_{s}+o_{L}=\frac{\varphi_{S}}{\varphi_{Sf}}+\frac{\varphi_{L}}{\varphi_{Lf}}=2$$

| $P^{3}=P^{3HN}+\frac{\varphi_{Sf}}{\varphi_{Sf}}P^{3S}+\frac{\varphi_{Lf}}{\varphi_{Lf}}P^{3L}+\frac{\varphi_{Sf}}{\varphi_{Sf}}\frac{\varphi_{Lf}}{\varphi_{Lf}}P^{3I}$ | Eqn (S10) |
| --- | --- |

| $P^{3}=\left( \varphi_{Sf}+\varphi_{Lf} \right)P^{3HN}+\frac{\varphi_{Sf}}{\varphi_{Sf}}P^{3S}+\frac{\varphi_{Lf}}{\varphi_{Lf}}P^{3L}+\frac{\varphi_{Sf}}{\varphi_{Sf}}\frac{\varphi_{Lf}}{\varphi_{Lf}}P^{3I}$ | Eqn (S11) |
| --- | --- |

| $P^{3}= \varphi_{Sf}\left( P^{3HN}+\frac{1}{\varphi_{Sf}}P^{3S} \right)+\varphi_{Lf}\left( P^{3HN}+\frac{1}{\varphi_{Lf}}P^{3L} \right)+P^{3I}$ | Eqn (S12) |
| --- | --- |

Property:

| $P^{2}=\varphi_{Sf}P^{2S}+\varphi_{Lf}P^{2L}$ | Eqn (S1) |
| --- | --- |

At full occupancy: $P^{2}=P^{3}$

| $P^{2S}=P^{3HN}+\frac{1}{\varphi_{Sf}}P^{3S}$ | Eqn (S13) |
| --- | --- |
| $P^{2L}=P^{3HN}+\frac{1}{\varphi_{Lf}}P^{3L}$ | Eqn (S14) |
| $P^{3I}\approx0$ | Eqn (S15) |

Binary mixture model origins from the rule of mixture, which can be applied to all composite material. Once the property values are evaluated from the constituent material aspect, the property values of composite material can be calculated. In other words, the coefficients (P^2S^ and P^2L^) in the binary mixture model are evaluated from the cage aspect.

However, the ternary mixture model is designed for the gas hydrates system. It considers the structural feature of gas hydrate. The hydrogen-bonded network permanently exists regardless of the presence of guest molecules. The small and large cages are embedded in the hydrogen-bonded network. This is different from the constituent material in the binary mixture model, which does not have a basic structural skeleton. P^3S^ and P^3L^ are the effects estimated from the lattice aspect, originating from the occupied small or large cages.

At full occupancy, which is a special condition, the binary and ternary mixture models should generate the same results. Then the Eqns (S13-S15) shows the relationship between the properties from the binary model and the ternary model properties.

To summarize, there is no conflict between the binary and ternary mixture models. The ternary mixture model is the updated version of the binary model, which considers the structural feature of gas hydrate. At full occupancy, ensuring two models will give the same results, Eqns (S13-S15) need to be satisfied. Under other conditions, only ternary mixture models can be applied.

**SI 2: Steepest ascent and steepest descent curve derivation**

This section will provide detailed information for the derivations of Eqn (3-5) in the Results and Discussion section of the manuscript

The relationship between the compressive stability limits and the small and larges’ occupancy can be expressed in analytical and numerical ways, as shown in Eqn (S16-S17) which are the same as Eqn (1-2) in the manuscript. The four coefficients a, b, c and d are equal to 4.6, 0.3, 1.7 and 0.7, respectively.

| $S \left( X,Y \right)=a+bX+cY+dXY$ | Eqn (S16) |
| --- | --- |
| $S (GPa)=4.6+0.3\times Q_{s}+1.7\times Q_{l}+0.7\times$ $Q_{s}$ $Q_{l}$ | Eqn (S17) |

**Steepest Descent/Ascent Curves**

**Step 1: take partial derivatives of** $\mathbf{S(X,Y)}$**:**

| $\frac{\partial S}{\partial X}=b+dY$ | Eqn (S18) |
| --- | --- |
| $\frac{\partial S}{\partial Y}=c+dX$ | Eqn (S19) |

**Step 2: Apply standard calculus:**

| $\frac{dY}{dX}=\frac{\frac{\partial S}{\partial Y}}{\frac{\partial S}{\partial X}}$ | Eqn (S20) |
| --- | --- |

| $\frac{\partial S}{\partial X} dY=\frac{\partial S}{\partial Y} dX$ | Eqn (S21) |
| --- | --- |

| $\left( b+dY \right)dY=\left( c+dX \right)dX$ | Eqn (S22) |
| --- | --- |

**Step 3: Integrate both sides:**

| $bY+\frac{d}{2}Y^{2}=cX+\frac{d}{2}X^{2}+K$ | Eqn (S23) |
| --- | --- |

Where K is a constant, has a unit of GPa, the same as $S$:

| $bY+\frac{d}{2}Y^{2}-\left( cX+\frac{d}{2}X^{2} \right)=K$ | Eqn (S24) |
| --- | --- |

Plug in the coefficient values

| $0.7Y+\frac{1.7}{2}Y^{2}-\left( 0.3X+\frac{1.7}{2}X^{2} \right)=K$ | Eqn (S25) |
| --- | --- |

The **Error! Reference source not found.**) shown above is the Eqn (3) in the manuscript, where $X$ is $Q_{s}$ and $Y$ is $Q_{l}$.

| $bY+\frac{d}{2}Y^{2}-\underset{R}{\underbrace{\left( cX+\frac{d}{2}X^{2} \right)}}=K$ | Eqn (S26) |
| --- | --- |

Use $R$ to replace $\left( cX+\frac{d}{2}X^{2} \right)$

| $bY+\frac{d}{2}Y^{2}-\left( R+K \right)=0$ | Eqn (S27) |
| --- | --- |

| $Y=\frac{-b\pm\sqrt{b^{2}+4\times\frac{d}{2}\times\left( R+K \right)}}{d}$ | Eqn (S28) |
| --- | --- |

**Considering the value of Y would be positive**

| $Y(X,K)=\frac{-b+\sqrt{b^{2}+2d\left( cX+\frac{d}{2}X^{2}+K \right)}}{d}$ | Eqn (S29) |
| --- | --- |

**Steepest ascent curve (starting from** $\boldsymbol{X=Y=0}$**)**

**Step 1: to calculate the** $\boldsymbol{K}$ **value for the steepest curve through** $\boldsymbol{x=y=0}$**, plug** $\boldsymbol{x=y=0}$ **into the equation**

| $0=\frac{-b+\sqrt{b^{2}+2d\left( c0+\frac{d}{2}0^{2}+K \right)}}{d}$ | Eqn (S30) |
| --- | --- |

$$\boldsymbol{0=}\frac{-b+\sqrt{b^{2}+2dK}}{d}$$

$$-b+\sqrt{b^{2}+2dK}=0$$

By solving **Error! Reference source not found.**), we can obtain the value of K.

$$\boldsymbol{K=0}$$

**Thus** $\boldsymbol{K=0}$ **for the steepest ascent curve.**

| $bY+\frac{d}{2}Y^{2}-\left( cX+\frac{d}{2}X^{2} \right)=0$ | Eqn (S31) |
| --- | --- |

**Step 2: find the ending point of the steepest ascent curve where** $\boldsymbol{Y=1}$**.**

| $b\times1+\frac{d}{2}\times1^{2}-\left( cX+\frac{d}{2}X^{2} \right)=0$ | Eqn (S32) |
| --- | --- |

| $\frac{d}{2}X^{2}+cX-\left( b+\frac{d}{2} \right)=0$ | Eqn (S33) |
| --- | --- |

| $X=\frac{-c\pm\sqrt{c^{2}+4\frac{d}{2}\left( b+\frac{d}{2} \right)}}{d}$ | Eqn (S34) |
| --- | --- |

Considering $X$ would be a positive value,

| $X=\frac{-c+\sqrt{c^{2}+4\frac{d}{2}\left( b+\frac{d}{2} \right)}}{d}$ | Eqn (S35) |
| --- | --- |

| $\boldsymbol{X=}\frac{-c+\sqrt{c^{2}+d^{2}+2db}}{d}$ | Eqn (S36) |
| --- | --- |

Step 3: Evaluate the Stability limit value of the ending point of the steepest ascent curve.

| $S_{max}\left( X=1, Y=1 \right)=a+b+c+d$ | Eqn (S37) |
| --- | --- |
| $S_{min}\left( X=0, Y=0 \right)=a$ | Eqn (S38) |

| $S\left( X=\frac{-c+\sqrt{c^{2}+d^{2}+2db}}{d}, Y=1 \right)=a+b\frac{-c+\sqrt{c^{2}+d^{2}+2db}}{d}+c+d\frac{-c+\sqrt{c^{2}+d^{2}+2db}}{d}$ | |
| --- | --- |
|  | Eqn (S39) |

| $S\left( X=\frac{-c+\sqrt{c^{2}+d^{2}+2db}}{d}, Y=1 \right)=a+c+\frac{-c+\sqrt{c^{2}+d^{2}+2db}}{d}\left( b+d \right)$ | |
| --- | --- |
|  | Eqn (S40) |

| $S\left( X=\frac{-c+\sqrt{c^{2}+d^{2}+2db}}{d}, Y=1 \right)=S_{max}-\left( 1-\frac{-c+\sqrt{c^{2}+d^{2}+2db}}{d} \right)\left( b+d \right)$ | |
| --- | --- |
|  | Eqn (S41) |

**Which is the same as Eqn (4) in the manuscript**

**Steepest decent curve (starting from** $\boldsymbol{X=Y=1}$**)**

**Step 1: to calculate the** $\boldsymbol{K}$ **value for the steepest curve through** $\boldsymbol{x=y=1}$**, plug** $\boldsymbol{x=y=1}$ **into the equation**

| $1=\frac{-b+\sqrt{b^{2}+2d\left( c+\frac{d}{2}+K \right)}}{d}$ | Eqn (S42) |
| --- | --- |

$$\boldsymbol{b+d=}\sqrt{b^{2}+2dc+d^{2}+2dK}$$

$$\boldsymbol{K=b-c}$$

**Thus** $\boldsymbol{K=b-c}$ **for the steepest decent curve.**

| $bY+\frac{d}{2}Y^{2}-\left( cX+\frac{d}{2}X^{2} \right)=b-c$ | Eqn (S43) |
| --- | --- |

**Step 2: find the ending point of the steepest decent curve where** $\boldsymbol{Y=0}$**.**

| $b\times0+\frac{d}{2}\times0^{2}-\left( cX+\frac{d}{2}X^{2} \right)=b-c$ | Eqn (S44) |
| --- | --- |

| $\frac{d}{2}X^{2}+cX+\left( b-c \right)=0$ | Eqn (S45) |
| --- | --- |

| $X=\frac{-c\pm\sqrt{c^{2}-4\frac{d}{2}\left( b-c \right)}}{d}$ | Eqn (S46) |
| --- | --- |

**Considering** $\boldsymbol{X}$ **is a positive value**

| $X=\frac{-c+\sqrt{c^{2}-2d\left( b-c \right)}}{d}$ | Eqn (S47) |
| --- | --- |

**Step 3: Evaluate the Stability limit value of the ending point of the steepest decent curve.**

| $S\left( X=\frac{-c+\sqrt{c^{2}-4\frac{d}{2}\left( b-c \right)}}{d}, Y=0 \right)=a+b\frac{-c+\sqrt{c^{2}-2d\left( b-c \right)}}{d}$ | Eqn (S48) |
| --- | --- |

| $S\left( X=\frac{-c+\sqrt{c^{2}-4\frac{d}{2}\left( b-c \right)}}{d}, Y=0 \right)=S_{min}+b\frac{-c+\sqrt{c^{2}-2d\left( b-c \right)}}{d}$ | Eqn (S49) |
| --- | --- |

Which is the same as Eqn (5) in the manuscript
